# Green synthesis of silver nanoparticles by *Kocuria flava* isolated from photovoltaic panels for combating infections, cancer, and water pollution

**DOI:** 10.3389/fmicb.2025.1658320

**Published:** 2025-10-30

**Authors:** Vishal Prajapati, Dhanusha Panchal, Manisha Dikshit, Rajesh Patel, Sunil Bhavsar, Ashish Patel, Dipak Kumar Sahoo, Kuldeep Luhana

**Affiliations:** ^1^Department of Life Sciences, Hemchandracharya North Gujarat University, Patan, Gujarat, India; ^2^GTU-School of Applied Sciences and Technology, Gujarat Technological University, Ahmedabad, Gujarat, India; ^3^GTU-School of Pharmacy, Gujarat Technological University, Ahmedabad, Gujarat, India; ^4^Department of Veterinary Clinical Sciences, College of Veterinary Medicine, Iowa State University, Ames, IA, United States

**Keywords:** rooftop solar panel, *Kocuria flava*, silver nanoparticles biosynthesis, environmental and biotherapeutic activity, sustainable development goals

## Abstract

The extremophilic bacterial community associated with photovoltaic solar panels has demonstrated significant resilience to harsh environmental conditions such as desiccation, thermal fluctuations, and intense solar exposure. The bacterial strain *Kocuria flava* KKYHNGU1 was isolated from a PV panel and characterized for its resistance to alkaline pH (pH 9), salinity (1–9% w/v), UV radiation (approximately 8 min), and high temperatures (up to 55 °C). Bio-manufactured AgNPs from this isolate were characterized, including UV–visible spectroscopy (with wavelength peaks at 437 nm), FTIR, and X-ray diffraction, validating the crystalline configuration of the AgNPs, exhibiting intensity peaks at 28.02°, 32.46°, and 46.36°. The spherical shape and size (96 nm diameter) along with the silver content (87.06% w/w) of bio-synthesized AgNPs was confirmed through scanning electron microscopy coupled with energy-dispersive X-ray spectroscopy. Metabolomic analysis through LC-MS revealed the presence of bioactive compounds possessing significant antimicrobial and anti-cancer properties, in addition to marked reduction capabilities. They have manifested notable antibacterial potential against *Staphylococcus aureus* with a 14 mm zone of inhibition and also proved effectiveness against *Serratia marcescens*, *Bacillus cereus*, and *Escherichia coli*. Additionally, the AgNPs effectively removed trimethoprim, achieving a 63.42% removal at 10 ppm within 120 min. These bio-fabricated AgNPs displayed remarkable anti-proliferative and pro-apoptotic activity against the THP-1 monocytic leukemia cell line, yielding an IC50 measurement of 40 μg/mL. These findings underscore the prospective applications of biogenic AgNPs in wastewater treatment and biomedical fields, illustrating their multiple functionalities.

## 1 Introduction

The bacteriological green synthesis of silver nanoparticles (AgNPs) is a promising, cost-effective, and environmentally friendly approach in nanobiotechnology. This process leverages the ability of bacteria to reduce silver ions (Ag^+^) to produce nanoparticles with unique properties, including antimicrobial, anticancer, and bioremediation efficiency (Dayma et al., 2024). This biogenic method is superior to traditional chemical and physical synthesis because it avoids hazardous chemicals and offers biocompatibility (Dhaka et al., 2023; Huq et al., 2023; Singh et al., 2015). This aligns with the broader scientific trend of using biological systems for their environmental advantages and the unique characteristics they impart to the resulting nanoparticles (Junejo et al., 2024; Manisekaran et al., 2024; Renjusha and Kumar, 2023).

Bacteria in the Bacillus genus, such as *Bacillus mojavensis* and *Bacillus pumilus*, produce biocatalysts and secondary metabolites that act as reducing and capping agents, ensuring the synthesis of stable and uniform nanoparticles (Iqtedar et al., 2019; Mahmoud et al., 2016). Additionally, the use of bacterial biomass or cell-free extracts simplifies the process, as extracellular synthesis is favoured over intracellular production for easier nanoparticle isolation (Saritha and Prabha, 2022; Singh and Mijakovic, 2022).

Rooftop photovoltaic solar panels host diverse microbial communities adapted to extreme conditions like high solar radiation, temperature fluctuations, and desiccation (Luhana et al., 2025; Moura et al., 2021). Cyanobacterial genus *Chroococcidiopsis* isolated from solar panels in Valencia, Spain, and exhibited resistance to desiccation and UV-C radiation, suggesting potential applications in astrobiology and biotechnology (Baldanta et al., 2023). Stress-resistant genera like *Hymenobacter* and *Deinococcus* are prevalent (Porcar et al., 2018; Tanner et al., 2020). Majority of these bacteria are capable of producing pigments like carotenoids, which imparts antioxidant properties, making them candidates for novel antioxidant applications (Luhana et al., 2025; Tanner et al., 2019). The microbial colonization of solar panels is subjected to environmental factors and fluctuations affecting species richness and diversity (Tanner et al., 2020). Biofilm-producing bacteria on solar panels can impact system efficiency through soiling (Ortiz et al., 2024). Despite this, these panels are a promising source of microorganisms with genes for novel extremozymes, pigments, and secondary metabolites, particularly those involved in stress resistance and biofilm formation (Jotta et al., 2024; Ortiz et al., 2024). The functional profiles of these bacterial communities are consistent across different geographical zones, highlighting their adaptability and opening new dimensions for biotechnological applications (Dorado-Morales et al., 2016; Tanner et al., 2018). Overall, the study of microbial diversity on solar panels enriches our knowledge and understanding of microbial life growing under extreme conditions and opens new dimensions for manipulating these organisms for various biotechnological applications.

*Kocuria flava* derived silver nanoparticles represent a promising approach in green synthesis. A study on a marine isolate of *K. flava* focused on using its extracellular metabolites to synthesize and characterize AgNPs. These nanoparticles showed strong antibacterial activity, particularly a synergistic effect with the antibiotic ciprofloxacin against multi-drug resistant strains like *P. aeruginosa* and *E. coli* (Farhan and Shareef, 2024). The integration of this bacteriogenic synthesis with traditional and advanced applications of AgNPs could lead to advancements in nanomedicine (Mohanata et al., 2023).

The characterization of bacteriologically produced silver nanoparticles (AgNPs) is crucial for understanding their properties. Various analytical tools are used, including UV–vis spectroscopy to confirm AgNP formation (Deljou and Goudarzi, 2016; Wypij et al., 2021). SEM and TEM confirm their size (typically 5–50 nm) and shape (most often spherical) (El-Naggar et al., 2024). Additionally, Fourier transform infrared spectroscopy is used to identify the biomolecules responsible for capping and stabilization (Jain et al., 2021), while X-ray diffraction and dynamic light scattering confirm their crystalline properties, size distribution, and stability (Rosman et al., 2020).

The biotechnological application of synthesized AgNPs largely depends on the bacteria used for production. For instance, a consortium of *Lactobacillus* sp. and *Bacillus* sp. synthesized AgNPs (4.65 to 22.8 nm) with significant antimicrobial efficiency against multi-drug resistant *S. aureus* and *P. aeruginosa* (Al-Asbahi et al., 2024). Similarly, AgNPs from *Klebsiella pneumoniae*, *Micrococcus luteus*, and *Enterobacter aerogenes* were effective in bioremediation and antimicrobial applications (Patel et al., 2024). *Klebsiella pneumoniae* mediated synthesis of AgNPs also demonstrated efficient dye removal and antibacterial activities (Desai et al., 2024). In another study, AgNPs (82.76 nm) from *Pseudomonas otitidis* showed moderate antibacterial and antibiofilm activities (Jose et al., 2024). *Geobacillus* spp. synthesized AgNPs, mostly under 100 nm, that were spherical and stable (Cekuolyte et al., 2023). Other studies have shown that *Paenibacillus* sp. can promptly synthesize AgNPs (15–55 nm) with bactericidal activities against *Salmonella enteritidis* and *Candida albicans* (Huq et al., 2023). Together, these studies highlight the versatility of bacterial sources for the biomanufacturing of AgNPs, offering a sustainable alternative to conventional methods and opening up new avenues for applications in anti-cancer therapy, antimicrobial activity, and bioremediation aligning with SDGs.

The immense potential of green-synthesized AgNPs lies in their diverse applications. In antimicrobial therapy (Deljou and Goudarzi, 2016). AgNPs are effective against pathogenic strains like *S. aureus* (MRSA) and *P. aeruginosa* by disrupting cell membranes, blocking DNA replication, and producing reactive oxygen species (ROS) (Huq et al., 2023; Mahmoud et al., 2016). Furthermore, they show promise in cancer treatment, inducing apoptosis in cancerous cells and enhancing the efficacy of chemotherapeutic drugs (Sintubin et al., 2012; Wypij, 2021).

AgNPs also have potential in agricultural applications, where they have been shown to enhance seed germination and protect crops from bacterial diseases (Singh et al., 2015). Their bioremediation efficiency is also significant, as they can facilitate the degradation of organic and inorganic pollutants (Ibrahim et al., 2021; Yamini and Rajeswari, 2023). Additionally, due to their biocompatibility and unique optical properties, AgNPs are being used in biosensors, drug delivery, and wound healing (Dhaka et al., 2023; Paterlini et al., 2021).

While AgNPs have been biosynthesized using *K. flava* before, our study is the first to report isolation of a strain of *K. flava* from a photovoltaic panel which demonstrate a specific anti-cancer efficacy against blood cancer cell lines, antimicrobial potential, and also antibiotic removal from waste water. All the potential applications aligning with Sustainable Development Goals (SDGs) 6, 9, 11, and 13.

## 2 Materials and methods

### 2.1 Materials

N-agar and N-broth, Reasoner’s 2A agar, Marine agar (Zobell), Luria Bertani broth, Muller Hilton agar, RPMI-1640 cell culture media, Phosphate buffer saline (pH 7.4) and silver nitrate solution (AgNO_3,_ 99.9% purity) were procured from HIMEDIA (Mumbai, India). THP1 cell line was acquired from NCCS (Pune, India).

### 2.2 Sample collection and isolation of bacteria

**Figure 1:**
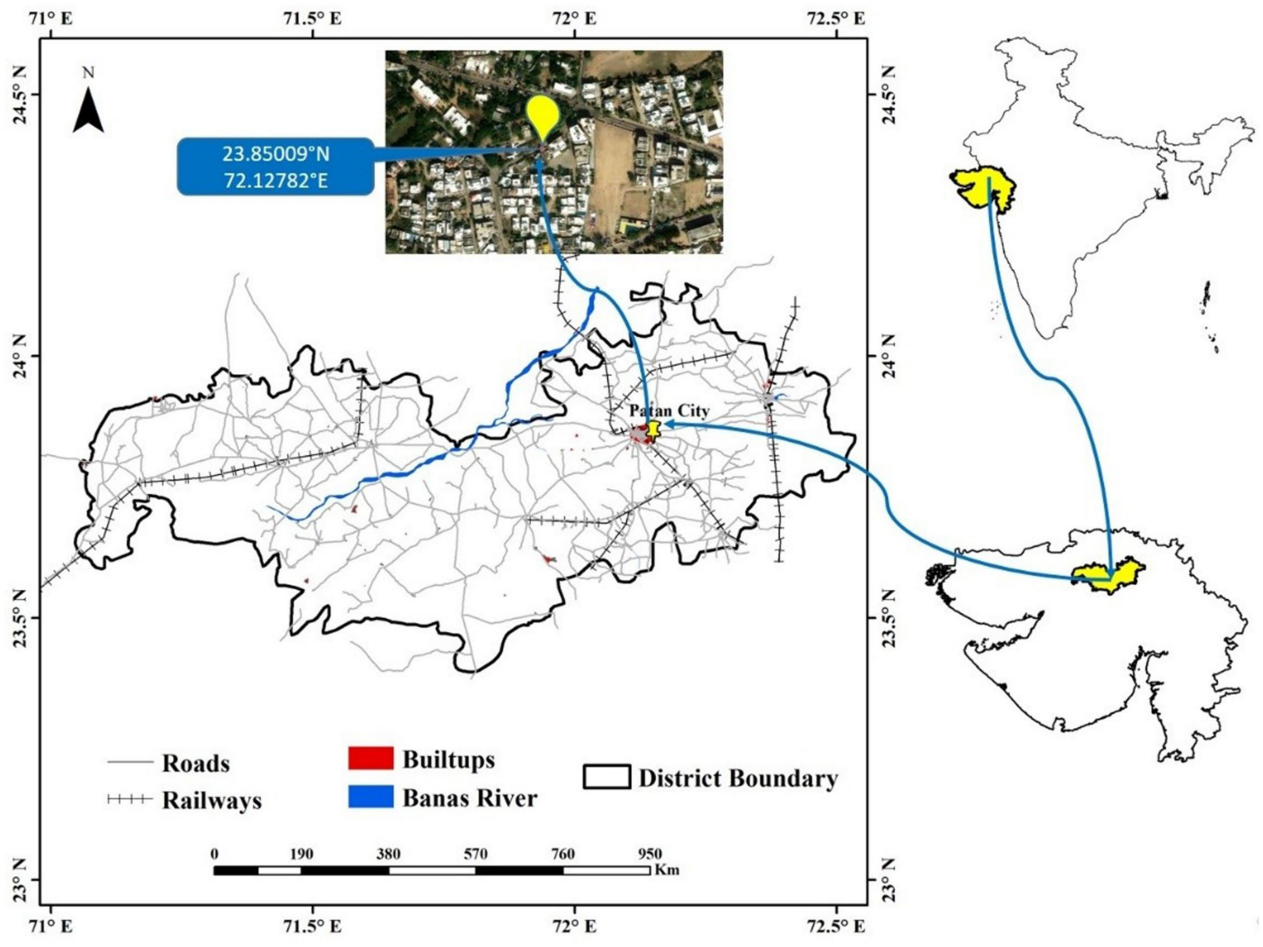
Sample collection site.

A scratch sample was obtained from the rooftop photovoltaic panel on the rooftop mounted on residential building in Patan, Gujarat, India (Figure 1), at 23.85009°N and 72.12782°E. To collect the scratch sample, a sterile PBS solution was poured onto the surface of the solar panel. Then, a sterile squeegee was used to firmly wipe the surface and gather the sample. Finally, approximately 40 mL slurry solution containing soil and dust was collected in a sterilized zip-lock bag. The samples were kept in portable ice chest and transported to the research laboratory for further processing. This was done in mid-summer during the month of May. During the sampling procedure, the sky was clear with moderate wind, atmospheric and rooftop photovoltaic surface temperatures were 38 and 47 °C, respectively, whereas the approximate relative humidity was around 30%. For further processing, the collected sample was serially diluted through standard procedure. The bacterial strain was isolated and screened on Luria Bertani agar, nutrient agar, Zobell marine agar, and Reasoner’s 2A agar, and incubated for 30 h at 37 °C.

To examine their adaptability under harsh conditions, several stress tests were performed on the isolated bacterial strains. These were to check their survival in different salt concentration, pH, heat, and UV radiation. The experimentation procedure was performed by plating on Luria Bertani agar with 15 μL of serially diluted culture (10^−5^). The resistance to pH was performed by modifying the growth medium to pH values of 4, 5, 6, 7.2 (as per normal media composition) and 8. To investigate salt tolerance the isolated cultures were subjected to grow in the culture media containing higher NaCl concentrations (1 to 9% w/v). The plates were incubated over night at different temperatures of 20, 30, 45, 50, 55, and 60 °C for evaluating the heat resistance capacity of the isolates. To examine the UV resistance potential of isolates the plates were subjected to UV radiation exposure for 30 s to 8 min just after seeding the plates.

### 2.3 16S rRNA based molecular identification of isolates

For molecular identification of the isolates, a set of forward primer (16SrRNA-F-27F-AGAGTTTGATCCTGGCTCAG) and reverse primer (16SrRNA-R-1492R-CGGTTACCTTGTTACGACTT) were used to amplify the 16S rRNA gene segment. The PCR amplicon was subjected for purification to remove any impurities. ABI 3730xl Genetic Analyzer and the BDT v3.1 cycle sequencing kit was employed for sequencing of amplified PCR product. Using final amplified PCR product of 1,201 bp sequence a phylogenetic tree was created to check the evolutionary relevance of the isolate. The sequence was subjected to NCBI BLASTn tool for identification of isolated bacterial strain and to retrieve the similar sequences from the GenBank database. The sequences were further allowed for sequence alignment using ClustalW program. For every aligned sequence, pairwise deletion method was applied for elimination of the ambiguous, and MEGA version 12.0 (Molecular Evolutionary Genetics Analysis) software tool was employed for establishment of phylogenetic relationships, which was developed using aligned sequences. This was accomplished by feeding the data to neighbor-joining (NJ) algorithm using Maximum Composite Likelihood evolutionary distances and the Kimura 2 parameter using more than 1,000 replicates (Bruno et al., 2000; Kumar et al., 2024).

### 2.4 LC-MS analysis of secondary metabolites

Secondary metabolites were extracted utilizing a solvent extraction methodology. Ethyl acetate served as the solvent for the extraction processes of secondary metabolites. To initiate the extraction, the bacterial isolate was cultured in LB broth and subjected to incubation at 37 °C for a period of 60 h at 150 rpm. Following the incubation period, Whatman filter paper was employed to filter the liquid culture. Subsequently, the resultant filtrate was combined with the solvent in a 1:1 volumetric ratio and permitted to repose for 5 min, facilitating the formation of two distinct immiscible layers. In follow up, the upper layer of the solvent, which contained the extracted components was separated through funnel. Finally, the solvent was evaporated with the help of a rotary vacuum evaporator, and the resultant substance was subjected to drying in order to procure the crude metabolite. The crude extract was dissolved in a designated solvent and conserved at a temperature of 4 °C. The sample was analyzed through liquid chromatography-mass spectrometry methodologies. A Shimadzu Prominence LC system equipped with an MS-compatible C18 reversed-phase column (Synergi-MAX-RP, 50 × 2 mm, 2 μm, 100, Phenomenex) was employed to execute high-performance liquid chromatography (HPLC). To avert any degradation, both the auto-sampler and the column were maintained at a temperature of 10 °C. The sample (5 μL) was injected into the column, and a low concentration of MTBE in MeOH was utilized to elute out the material at a flow rate of 0.4 mL/min. Four gradient conditions were implemented for the procedure: 0 min (100% MeOH), 10 min (50% MeOH), 12 min (25% MeOH), and 15 min (0% MeOH). A 4000 QTRAP hybrid triple quadrupole linear ion trap mass spectrometer (Applied Biosystems/Sciex) was directly connected to the column effluent (Kang and Kim, 2020).

### 2.5 Synthesis of AgNPs

**Figure 2:**
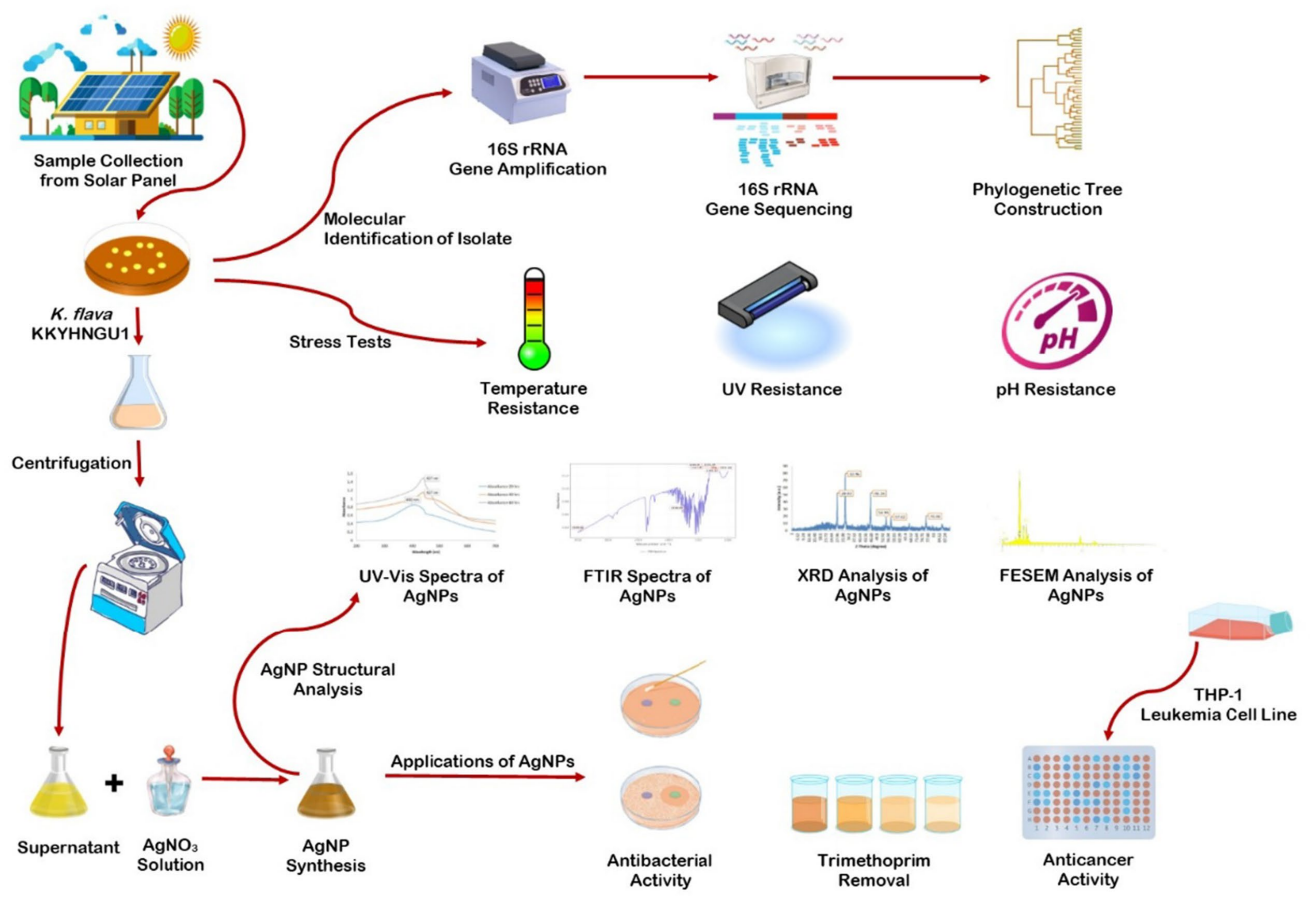
Graphical representation of steps followed in the AgNP bio-synthesis using *K. flava* KKYHNGU1.

*K. flava* KKYHNGU1 isolated and identified from rooftop solar panel was grown in liquid nutrient media for 24 h at 37 °C. Post incubation, the culture was allowed to centrifuge (15 min; 7,500 rpm) and the supernatant was collected for further processing. For silver nanoparticle production, the supernatant was mixed with 25 × 10^−3^ M AgNO_3_ solution in equal proportion and incubated for 60 h in the dark. Then it was allowed to centrifuge for 20 min at 11,000 rpm to pellet down the nanoparticles, which were washed with sterile double-distilled water and ethyl alcohol. Centrifugation was repeated using the same protocol and parameters. After 2–3 rinsing steps, the AgNPs were dried in oven at 65 °C for their downstream processing. The stages involved in the synthesis and application of AgNPs from *K. flava* KKYHNGU1 are shown in Figure 2.

### 2.6 Characterization of AgNPs

Synthesized AgNPs were subjected to number of analytical techniques including FTIR, XRD, UV–visible spectrophotometry, SEM, and EDX for characterization.

#### 2.6.1 UV–visible spectroscopic analysis

The existence of silver nanoparticles was confirmed through UV–visible spectroscopic analysis (LABMAN, LMPS-UV1900S) with 1 nm resolution in the range of 200–800 nm (Luhana et al., 2025).

#### 2.6.2 FT-IR spectrometric analysis

Identification of functional and reactive moiety coated on AgNP surface was carried out by FTIR (Bruker) spectroscopic analysis in the wave number range of 1,000–3,500 cm^−1^ (Desai et al., 2024).

#### 2.6.3 X-ray diffraction analysis

For X-ray diffraction pattern, a Rigaku Miniflex 600 (Phaser XRD-D2 powder X-ray diffractometer) was used at the set parameters of 30 kV and 2 mA. The sample was analyzed with Cu-Kα radiation covering a extensive range of Bragg angles, *θ* (3° ≤ 2*θ* ≤ 90°). The data generated by XRD was compared with standard established data provided by Joint Committee on Powder Diffraction. Mean crystallite size was determined through XRD line broadening measurements with the help of Debye–Slanderer equation (Patel et al., 2024).

#### 2.6.4 Scanning electron microscopy coupled EDX

Final detailed structural parameters of synthesized silver nanoparticles were acquired through SEM. The sample was allowed to be in the high vacuum mode with energy supplied at 20 kV with working distance of 7 mm. The micrographic images revealed a uniform distribution of nanoscale nearly spherical particles in the range from 2 μm to 118 nm (FEI, APREO LoVac). EDX spectroscopy was used for confirmation of the w/w% of elements involved in bio-manufacturing AgNPs (Ag, C, O, N, P) (Desai et al., 2024).

### 2.7 Antibacterial activity of AgNPs

Agar disc diffusion protocol was used to quantify the bactericidal activity of the AgNPs. This was confirmed by analyzing the zone of inhibition (mm) against selected gram-positive bacteria like *Staphylococcus aureus* and *Bacillus cereus* along with gram-negative bacteria like *Escherichia coli* and *Serratia marcescens* on Muller Hilton agar medium. The nutrient medium was seeded with the selected bacterial cultures and then paper disc (0.5 cm diameter) which were pre-soaked and sonicated in AgNP solution having concentration of 1 mg, 2 mg, and 3 mg of AgNPs were placed in respective areas of plates. The plates were kept in incubator for 24 h at 37 °C to detect the zone of growth inhibition (mm).

### 2.8 Trimethoprim removal by AgNPs

To determine the adsorbent efficiency of AgNPs for antibiotic, the trimethoprim removal test was conducted using 500 mg of trimethoprim. AgNPs (5 mg) were mixed with different concentrations (10, 25, 50 ppm) of the trimethoprim and stirred at the speed of 400 rpm on magnetic stirrer. Readings were recorded in the range of 200–400 nm at every 20 min interval up to 180 min with the help of UV–vis spectrophotometer. The percentage removal efficiency was calculated with the help of following equation as shown below:


$$\%\text{removal}=\frac{\mathrm{OD}\;\mathrm{at}\;0\;\min-\mathrm{OD}\;\mathrm{at}\;\text{Interval}}{\mathrm{OD}\;\mathrm{at}\;0\;\min}\times 100$$

### 2.9 Anticancer activity of AgNPs

The monocytic leukaemia cell line THP1 was used to determine the cytotoxic efficiency of *K. flava* KKYHNGU1 derived AgNPs. The cells were allowed to grow in liquid media (RPMI-1640) and 10% FBS (foetal bovine serum) was also added as supplement (Luhana and Bariya, 2025). In each well of microtiter plate, 1 × 10^4^ THP1 cells in 50 μL growth medium were adjusted and kept in incubation for 24 h at 37 °C with 5% CO_2_ prior to treatment of AgNPs at concentrations of 10, 20, 30, 40, and 50 μg/mL. After incubation of 48 h, WST-8 staining was performed by the addition of 10 μL WST-8 and 90 μL of cell suspension was followed by 2–4 h of reaction. Dimethyl sulfoxide (DMSO) was added to dissolve the crystals produced and the absorbance was recorded at 450 nm through an ELISA reader (Biotek) (Luhana and Bariya, 2023).

## 3 Results

### 3.1 Isolation and identification of bacterial strain

**Figure 3:**
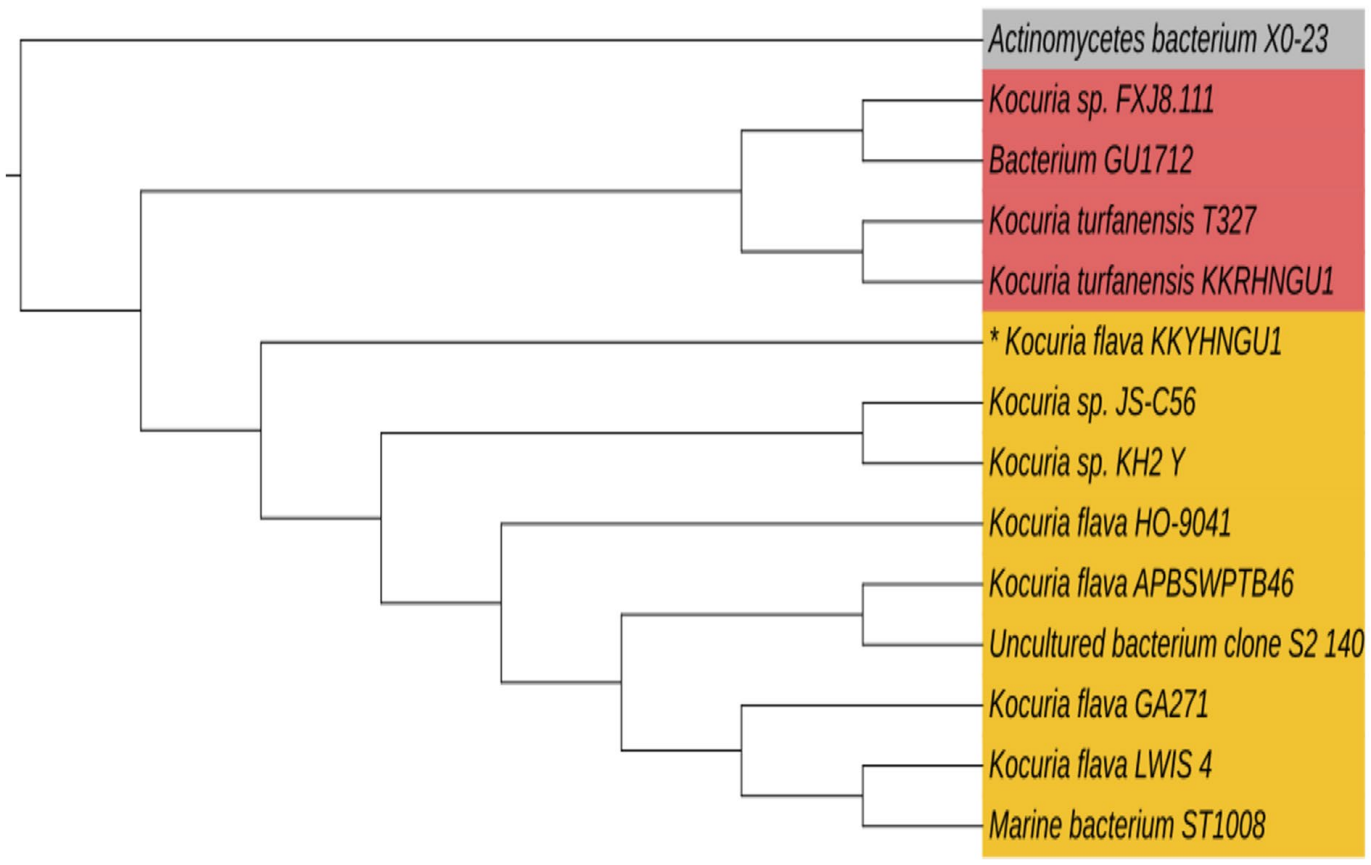
Phylogenetic tree constructed using 16S rRNA sequences of the photovoltaic solar panel isolate *Kocuria flava* KKYHNGU1. MEGA-12 neighbor-joining approach was used to create the tree.

The microorganism that was extracted from a solar rooftop panel has been successfully identified. This organism displayed a vibrant yellow-hued colony characterized by the presence of carotenoid pigments and demonstrated significant biofilm production capabilities. The organism had a coccoid morphology and was classified as gram-positive bacteria. The isolated bacterial strain was ultimately identified as *Kocuria flava* (KKYHNGU1) via 16S rRNA sequencing methodology (NCBI GenBank Accession—OR251787). The phylogenetic tree is depicted in Figure 3. *Kocuria flava* (KKYHNGU1) exhibited remarkable resistance to salinity levels ranging from 1 to 9% (w/v NaCl) and demonstrated resilience to prolonged exposure to ultraviolet radiation for durations of up to 8 min. The isolate exhibited sensitivity to highly acidic conditions with a pH of 3, yet it demonstrated enhanced growth at pH levels of 6, 8, and 9. Bacterial proliferation was noted at moderate mesophilic temperatures (30 °C, 37 °C) as well as elevated temperatures (45 °C, 50 °C, and 55 °C), although growth was not sustained at temperatures exceeding 55 °C.

### 3.2 LC-MS analysis

As shown in Table 1 LC-MS analysis indicated the existence of a variety of bioactive compounds. The identification of these compounds was done using the NIST database. On the basis of elevated *m*/*z* ratios, we identified the top five secondary metabolites, which encompass short peptides, antibacterial derivatives, and compounds featuring cyclical nature, all of which predominantly contributed to the reduction and so stabilization of silver nanoparticles.

**Table 1:** Identified secondary metabolites with potential role in AgNPs synthesis.

S. No.	Compound	Mol. formula	*m*/*z* ratio
1.	(4R)-4-[(2S)-2-({2-[(1S)-1-amino-2-methylbutyl]-4,5-dihydro-1,3-thiazol-5-yl}formamido)-4-methylpentanamido]-4-{[(1S)-1{[(3S,6R,9S,12R,15S,18R,21S)-18-(3-aminopropyl)-12-benzyl-15-(butan-2-yl)-3-(carbamoylmethyl)-6-(carboxymethyl)-9-(1H-imidazol-5-ylmethyl)-2,5,8,11,14,17,20-heptaoxo-1,4,7,10,13,16,19-heptaazacyclopentacosan-21-yl]carbamoyl}-2-methylbutyl]carbamoyl}butanoic acid	C_66_H_103_N_17_O_16_S	1422.75
2.	Efrotomycin	C_59_H_88_N_2_O_20_	1183.55
3.	1-{[7-(2-Amino-2-oxoethyl)-10-(3-amino-3-oxopropyl)-13-benzyl-16-(4-hydroxybenzyl)-6,9,12,15,18-pentaoxo-1,2-dithia-5,8,11,14,17-pentaazacycloicosan-4-yl]carbonyl}prolylarginylglycinamide acetate	C_48_H_72_N_14_O_16_S_2_	1183.48
4.	Pentopyranosyl-(1 → 4)-6-deoxyhexopyranosyl-(1 → 2)-1-O-{(3beta,16alpha)-16-hydroxy-3-[(6-methylhexopyranuronosyl)oxy]-28-oxoolean-12-en-28-yl}pentopyranose	C_53_H_84_O_22_	1095.53
5.	Tylvalosin derivative	C_53_H_87_NO_19_	1080.55

### 3.3 UV–visible spectroscopy

**Figure 4:**
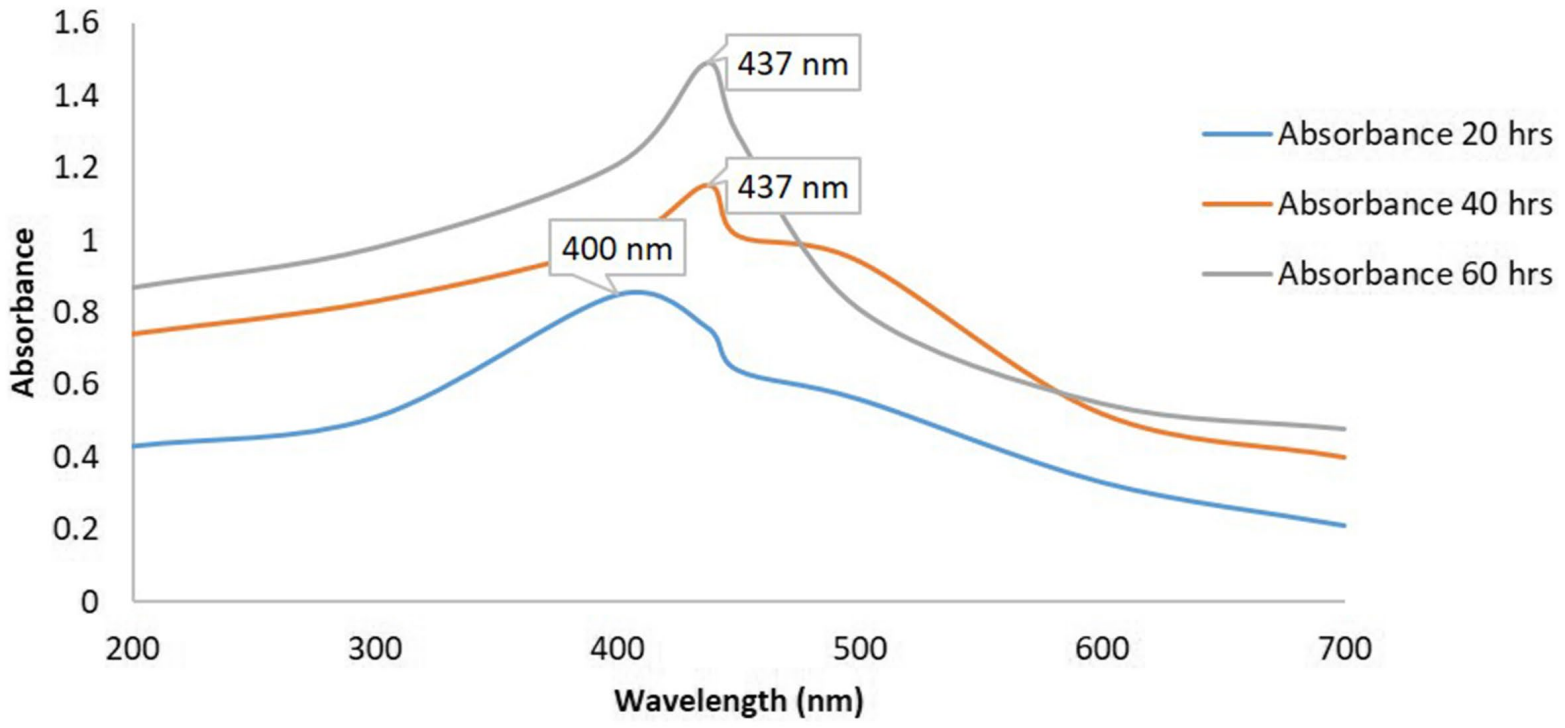
UV–vis spectra of AgNPs synthesized by *K. flava* KKYHNGU1.

The synthesis of silver nanoparticles (AgNPs) utilizing *K. flava* KKYHNGU1 was primarily validated through the observation of the colorimetric alteration of the reaction mixture, transitioning from yellow to brown following an incubation period of 60 h in a dark. As depicted in Figure 4, the absorption spectra corresponding to the AgNPs exhibited a wavelength range extending from 200 to 700 nm. The emergence of absorption peaks at 437 nm (Figure 4) was noted, with the broadness of the peak suggesting the presence of varying diameters of synthesized AgNPs.

### 3.4 FTIR analysis

**Figure 5:**
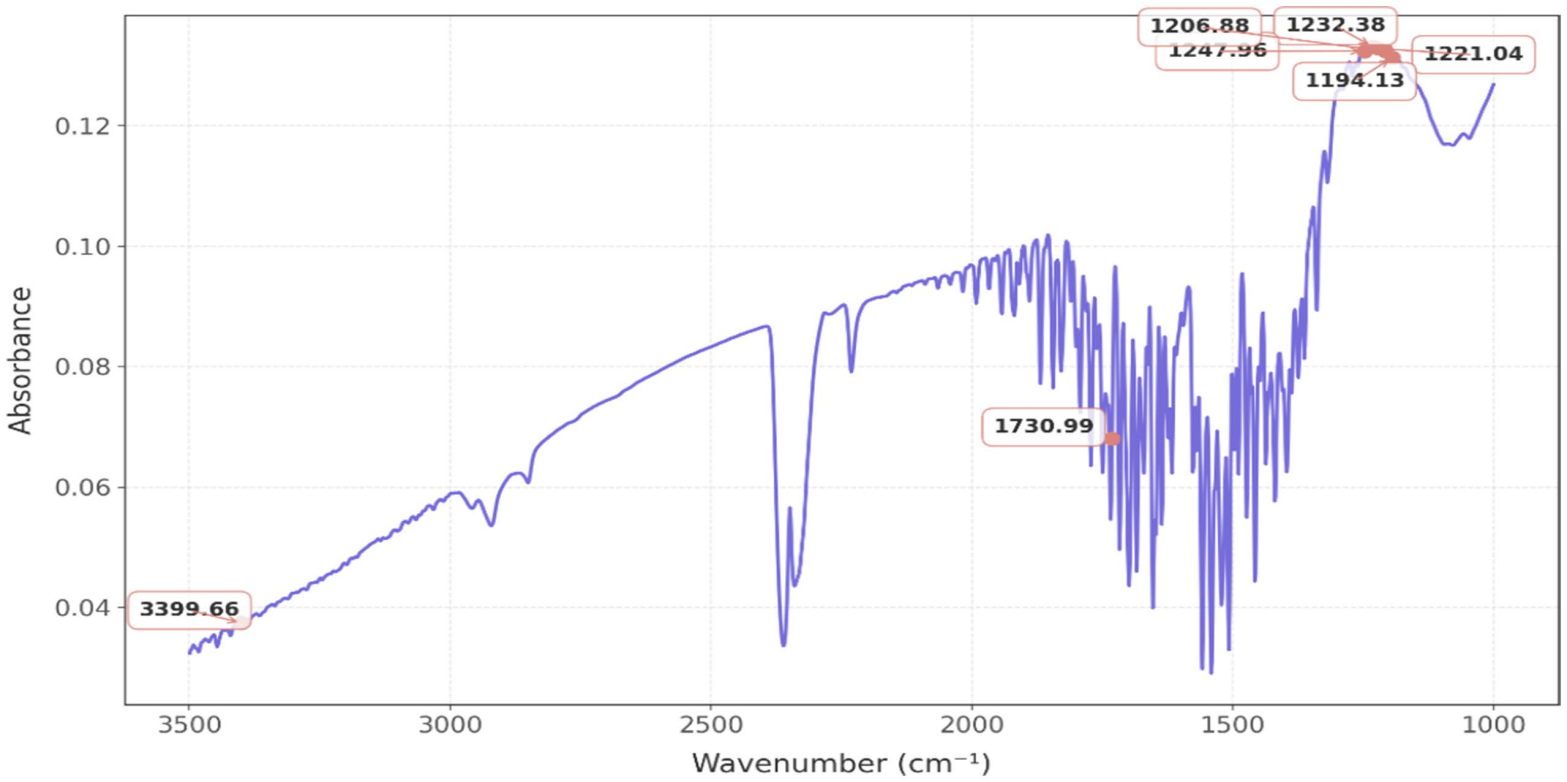
FTIR spectrum of AgNPs produced from *K. flava* KKYHNGU1 extract.

Figure 5 represents a classical FTIR spectrum, which reveals distinct spectral bands within the wavenumber range of 600 to 4,000 cm^−1^, predominantly observed at 1232.38 cm^−1^, 1221.08 cm^−1^, 1206.88 cm^−1^, and 1194.13 cm^−1^, indicative of C-O bond stretching, thereby suggesting the presence of esters and ethers; conversely, the peak at 1247.96 cm^−1^ implies the occurrence of C-N stretching, thus indicating the existence of amines. The peak at 1,730 cm^−1^ signifies the presence of C=O bonding, thereby substantiating the existence of esters, aldehydes, or ketones. The detection of an O-H stretch is corroborated by the presence of a peak at 3399.66 cm^−1^.

### 3.5 XRD analysis

**Figure 6:**
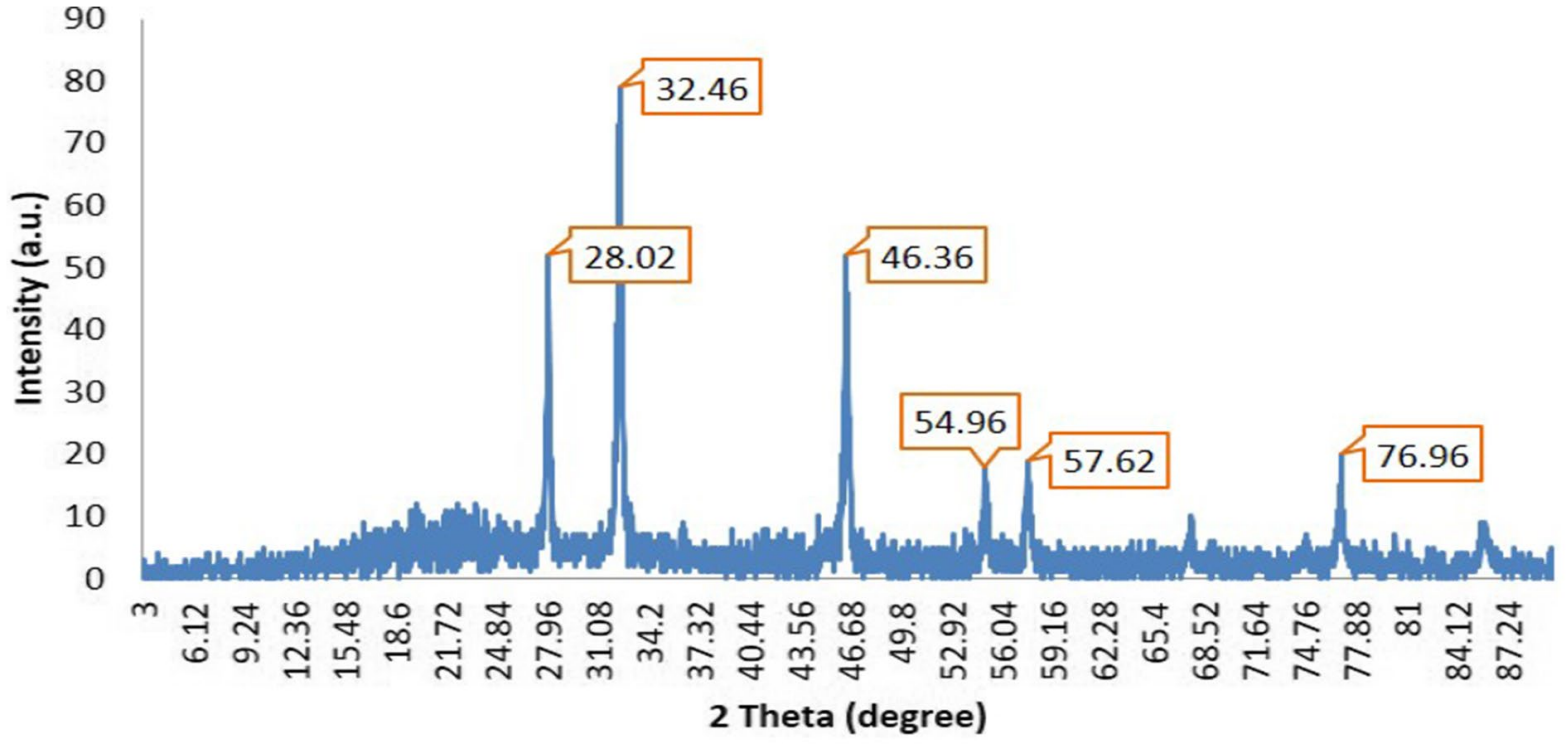
XRD analysis of AgNPs produced from *K. flava* KKYHNGU1 extract.

The X-ray diffraction analysis indicates the presence of prominent peaks at 28.02°, 32.46°, 46.36°, 54.96°, 57.626°, and 76.9°. Notably, this pattern exhibits three exceedingly pronounced sharp peaks at 28.02°, 32.46°, and 46.36°, with the peak at 32.46° exhibiting the highest intensity (Figure 6).

It is inferred that the synthesized silver nanoparticles possess a crystalline structure. These three distinct sharp peaks were employed in the application of the Scherrer equation to determine the parameters of the silver nanoparticles. Gaussian peak fitting techniques were utilized to ascertain the full width at half maximum (FWHM) and precise theta angle values. The determined crystallite size of the AgNPs was to be 25.9 nm.

### 3.6 FESEM-EDX

**Figure 7:**
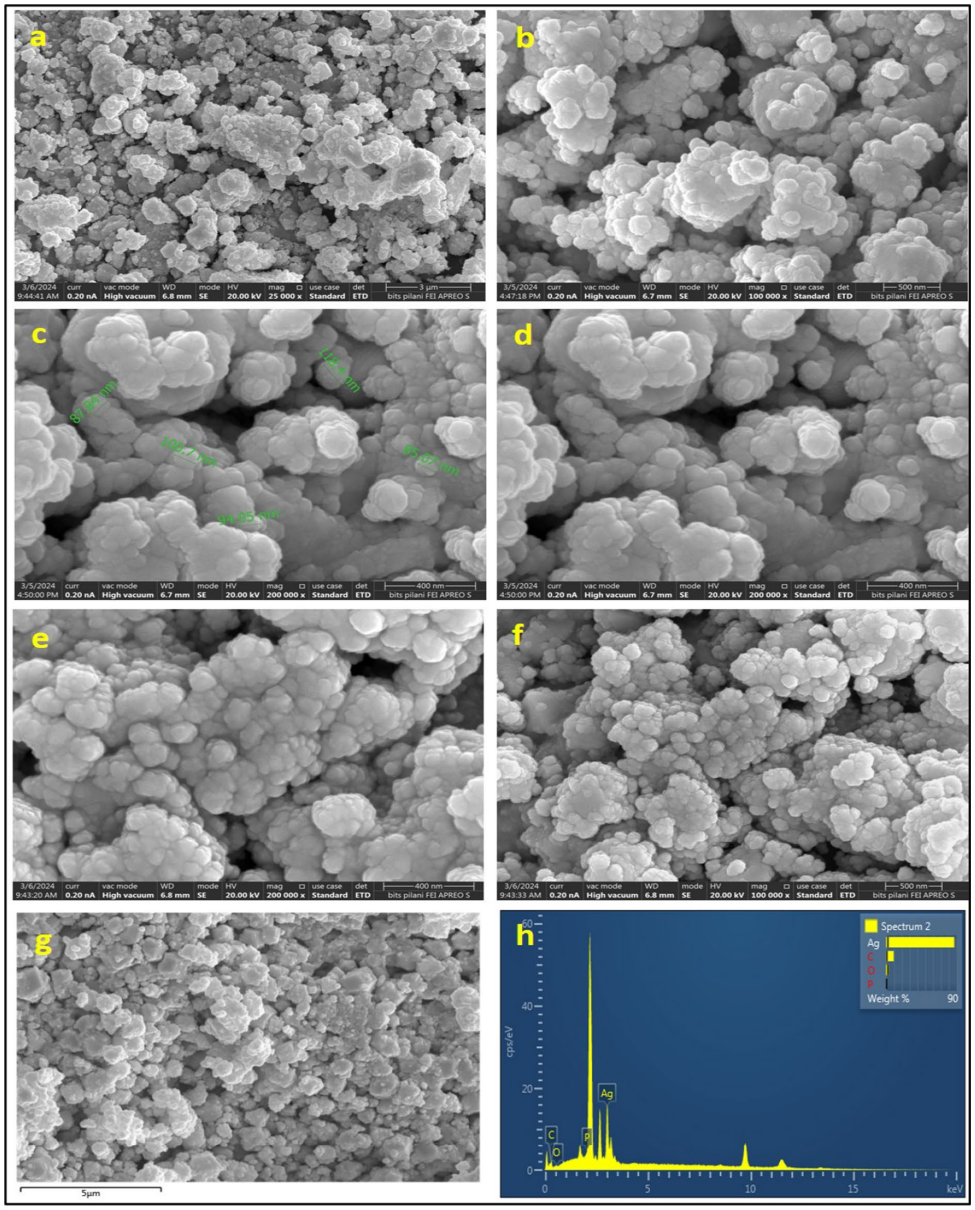
SEM micrographic images **(a–f)**, EDS spot **(g)**, and elemental graph **(h)** of AgNPs.

Scanning electron microscopy was used to analyze the shape and size of nanoparticles. The SEM imagery of the silver nanoparticles (AgNPs) is presented in Figures 7a–f at varying resolutions, revealing that the nanoparticles exhibited a predominantly spherical morphology and exhibited aggregation, resulting in the formation of a substantial structure. Figures 7c,d illustrates that the mean diameter of the NPs was determined to be 96 nm.

The spherical configuration of the AgNPs was further corroborated by energy dispersive spectroscopy (EDS) as depicted in Figure 7g, which displays the EDS spectrum for AgNPs, highlighting the presence of elemental peaks corresponding to C, O, P, and most notably Ag. Among all the elements involved in the synthesis of the NPs, silver was found to be the predominant constituent, with a weight percentage of 87.06%, followed by carbon at 9.69%, and oxygen at 3.25%. The high concentration of silver indicates the purity of manufactured AgNPs, while the occurrence of carbon is indicative of the biological origin of the NPs.

### 3.7 Antibacterial activity of silver nanoparticles

**Figure 8:**
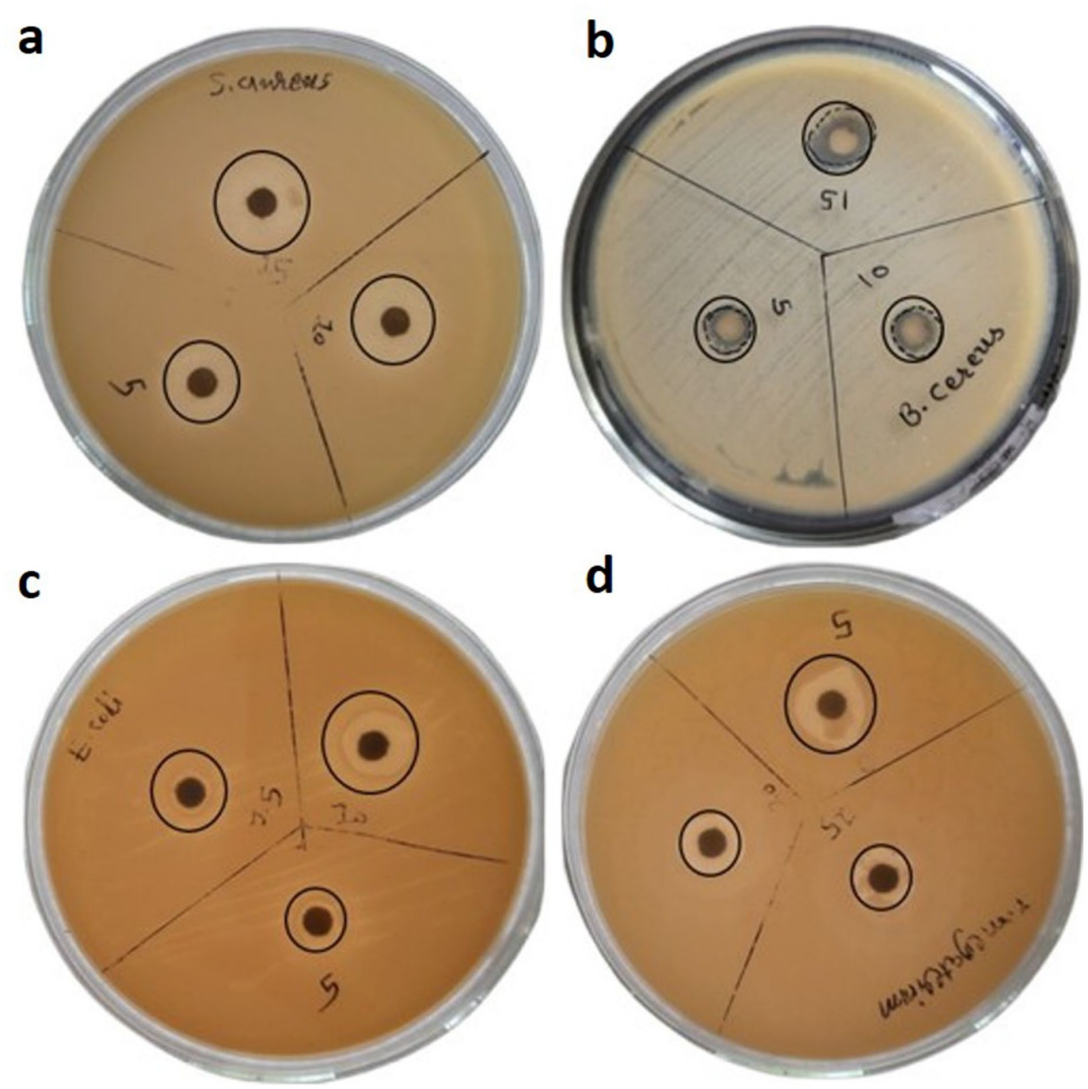
Antibacterial activity of AgNPs against bacterial strains of **(a)** *S. aureus* **(b)** *B. cereus* **(c)** *E. coli* and **(d)** *S. marcescens*.

Selected Gram-positive and Gram-negative bacterial strains were utilized to assess the antibacterial efficacy of the synthesized silver nanoparticles (AgNPs). The zone of inhibition measured (11 mm, 13 mm, 14 mm) was recorded against *S. aureus*, (8, 9, and 12 mm), against *B. cereus*, (9 mm, 11 mm, 12 mm) against *E. coli*, (5 mm, 7 mm, 9 mm) and against *S. marcescens*, (8 mm, 9 mm, 10 mm) at concentrations of 1, 2, and 3 mg of AgNPs, respectively (Figure 8).

### 3.8 Antibiotic trimethoprim removal by AgNPs

**Figure 9:**
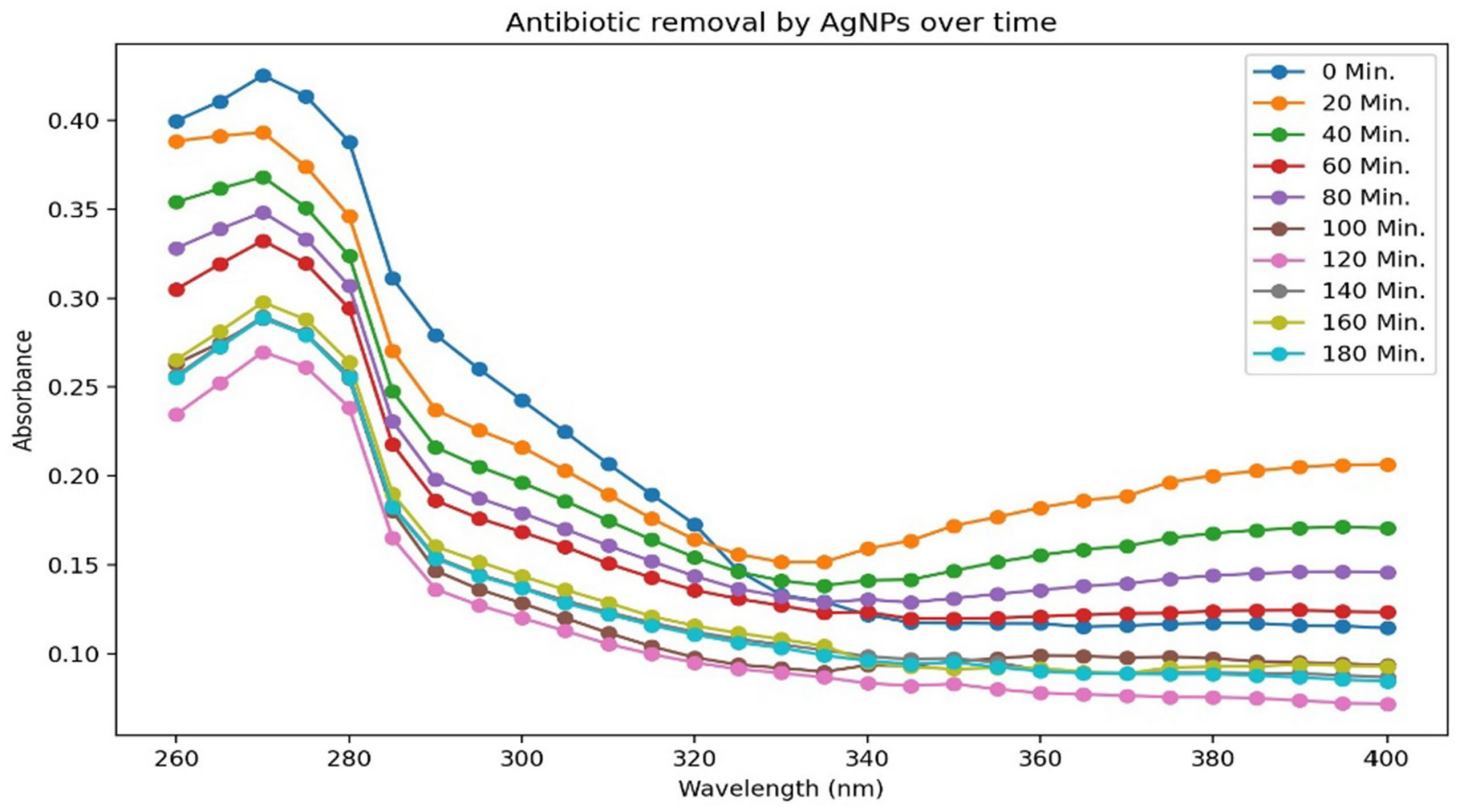
Antibiotic trimethoprim (20 ppm) removal at different time interval.

As shown in Figure 9, trimethoprim (TMP) exhibited the most pronounced peak of absorbance at a wavelength of 270 nm. In support of this observation, absorbance was quantified across the spectrum of 200 to 400 nm within the ultraviolet range. Figure 9 illustrates that the highest degree of removal attained was 63.42% after 120 min for a concentration of 10 ppm of AgNPs. These findings are encouraging for the decontamination of antibiotic residues from wastewater and contaminated soil.

### 3.9 Anti-proliferative activity

**Figure 10:**
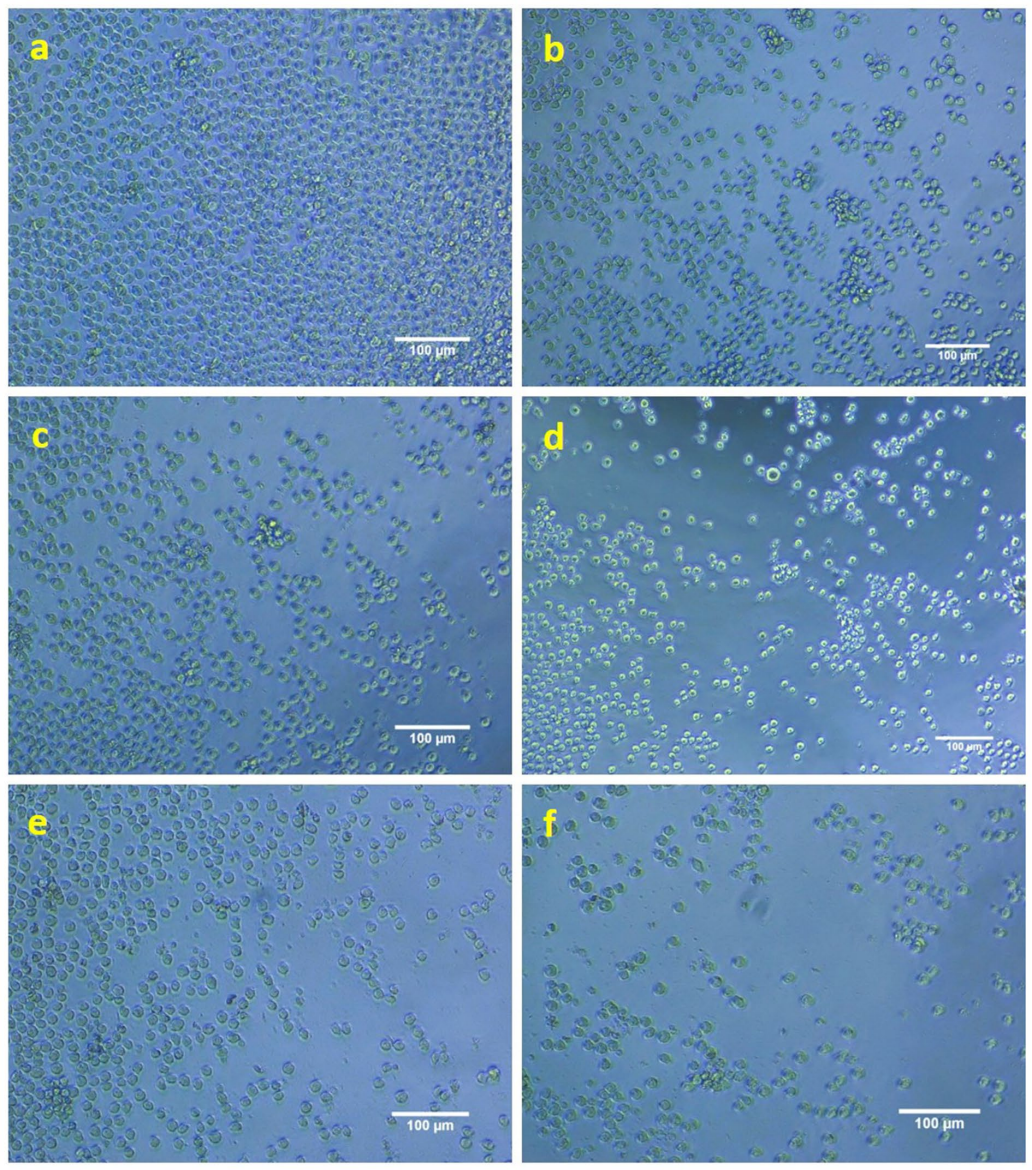
Anti-proliferative activity of AgNPs against THP1 cells (**a**—control; **b–f**—10, 20, 30, 40, and 50 mM concentration of AgNPs).

AgNPs have demonstrated significant anti-proliferative efficacy against the THP-1 monocytic leukemia cell line. A concentration of 40 μg/mL of AgNPs has shown the most pronounced anti-proliferative effect on the targeted cancer cells, corresponding to its IC50 value, which indicates the concentration required to induce cell death in 50% of the population. Figures 10b–f illustrates the enhanced anti-proliferative effects observed with varying concentrations of biosynthesized AgNPs when compared to the control presented in Figure 10a. These findings are noteworthy and underscore the potential therapeutic application of AgNPs derived from *K. flava* KKYHNGU1 in the treatment of hematological malignancies.

## 4 Discussion

This investigative inquiry focused on the microbial assemblage established on photovoltaic panels, wherein a distinct strain exhibiting a vibrant yellow colony and pronounced biofilm production capabilities was identified. Supporting evidence was provided by Tanner et al. (2019), who noted that numerous isolates derived from photovoltaic solar panels, particularly those cultured on marine agar (Zobell), exhibited pigmentation in hues of pink, red, and orange. In a similar vein, Dorado-Morales et al. (2016) discovered that a range of solar panel isolates cultivated using R2A agar displayed pink, red, orange and yellow pigmentation.

The bacterial isolate *K. flava* KKYHNGU1 has demonstrated its potential as a candidate for various applications due to its exceptional resistance properties, tolerating salt concentrations (w/v) between 1–9%, prolonged UV radiation exposure (8 min), alkaline condition (pH 9), and survival at temperatures reaching as high as 55 °C. Conversely, According to Dorado-Morales et al. (2016) a limited number of solar panel isolates exhibited resistance to extreme thermal conditions and acidic pH, while many of these isolates displayed the capability to endure elevated sodium chloride concentrations (up to 30% w/v) and substantial UV radiation. The influences of temperature and pH on the proliferation of *Chroococcidiopsis* spp. were also examined by Baldanta et al. (2023), reported that cultures experienced bleaching by the end of second day at about 50 °C, with no isolates demonstrating growth in liquid media. Furthermore, statistical evaluation revealed that although cultures maintained their original pigmentation green and remain alive long after 240 h (about 10 full days) at around 40 °C, growth exhibited significant reduction at both 4 °C as well as at 40 °C. All isolates demonstrated optimal growth at a pH of 7.5, which is near to physiological value.

Bacteriological bio-manufacturing of silver nanoparticles (AgNPs) entails the reduction and stabilization facilitated by functional groups of bacterial metabolites, including amino (–NH_2_), carboxyl (–COOH), and hydroxyl (–OH), which play roles of reducing agents for transformation of silver ions (Ag^+^) into elemental metallic silver (Ag0). Free amino acids and their derivative peptides containing functional groups, such as carboxylates, amines, thiols, and phosphates, are capable of adsorbing onto the AgNP surface, thereby mitigating nanoparticle agglomeration through the formation of stabilizing surface layer.

Specific arrangements of amino acid residues within peptides possess the capability to affect the morphology and dimensions of silver nanoparticles (Alsamhary, 2020; Wypij, 2021). As in the present study the secondary metabolites characterized through LCMS analysis, exemplified by “1-{[7-(2-Amino-2-oxoethyl)-10-(3-amino-3-oxopropyl)-13-benzyl-16-(4-hydroxybenzyl)-6,9,12,15,18-pentaoxo-1,2-dithia-5,8,11,14,17-pentaazacycloicosan-4-yl] carbonyl} prolylarginylglycinamide” exhibit disulphide linkages, as well as linear and cyclic peptide side chains, which collectively possibly enhance antimicrobial efficacy alongside mechanisms that disrupt cellular integrity leading to cell death. Furthermore, these compounds serve as proton donors, given that a multitude of functional groups possess the potential to engage in redox reactions. The presence of the compound “Pentopyranosyl-(1 → 4)-6- deoxyhexopyranosyl-(1 → 2)-1-O -{(3beta,16alpha)-16-hydroxy-3-[(6-methylhexopyranuronosyl) oxy]-28-oxoolean-12-en-28-yl}pentopyranose” also contributes to antioxidant and anticancer activity, attributed to the existence of certain triterpenoids and oligosaccharides. This is accomplished through the induction of oxidative stress within cells, ultimately leading to apoptotic processes. In both aforementioned compounds, the presence of side chain atoms bearing –COOH, –C=O, and –OH terminal groups is conducive to the reduction of silver ions, facilitating the synthesis of nanoparticles. Additionally, there exist derivatives such as “(4R)-4-[(2S)-2-({2-[(1S)-1-amino-2-methylbutyl]-4,5-dihydro-1,3-thiazol-5-yl}formamido)-4-methylpentanamido]-4-{[(1S) -1-{[(3S,6R,9S,12R,15S,18R,21S)-18-(3-aminopropyl)-12-benzyl-15-(butan-2-yl)-3-(carbamoylmethyl)-6-(carboxymethyl)-9-(1H- imidazol -5-ylmethyl) -2,5,8,11,14,17,20-heptaoxo-1,4,7,10,13,16,19-heptaazacyclopentacosan-21-yl]carbamoyl}-2-methylbutyl]carbamoyl} butanoic acid,” efrotomycin, and tylvalosin; all of which contribute to the antimicrobial properties of the synthesized silver nanoparticles. The chemical characteristics of these identified compounds are possibly playing crucial roles in nanoparticle synthesis and tentatively determining their potential applications for antimicrobial activity, antibiotic removal, and anticancer activity (Dayma et al., 2024).

The application of the *K. flava* KKYHNGU1 strain for the biosynthesis of AgNPs elucidated the efficacy of the isolated bacterial strain as a viable biological and potential agent in nanoparticle fabrication. The observed alterations correspond with characteristic attributes associated with the formation of AgNPs. It is possible that due to secondary metabolites reported earlier, *K. flava* KKYHNGU1 possesses the capacity to reduce Ag^+^ ions to AgNPs. The discernible absorption peaks signify the occurrence of nanoparticles of varying sizes or morphologies, gives a sign of mixed composition. Such discrepancies may arise from a multitude of biochemical, biophysical factors influencing the bio-fabrication process and reduction rate of silver ions. In a similar vein, AgNPs have been manufactured utilizing various bacterial isolates and strains, as documented in several studies.

Critical insights into the functional groups implicated in the synthesis of AgNPs by *K. flava* KKYHNGU1 are elucidated through FTIR spectrum examination and analysis. The biochemical processes occurring during the bacteriogenic production of silver nanoparticles are characterized by the detected molecular vibrations (bending and stretching).

Some reactive groups, including hydroxyl, carbonyls, and sulfhydryl were identified and may play a significant role in the stabilization and reduction of silver nanoparticles. The detection of bending vibrations (C–H and C–O), which are indicative of presence of proteins, peptides and amino acid structures and their derivatives, further substantiates the involvement of proteins in the synthesis process. It is highly probable that these functional groups are derived from proteins and amino acids synthesized by bacterial species that participate in the reduction process. In a similar vein, You et al. (2024) noted that Fourier transform infrared (FTIR) spectroscopy revealed spectral bands within the range of 500–4,000 cm^−1^. The stretching vibrations associated with alkanes (C–H), alcohols (O–H), primary amines (N–H), cyano (C–N), and carbonyl group (C–O) were recorded at 3,550 cm^−1^, 3,062 cm^−1^, 2,945 cm^−1^, 2,358 cm^−1^, 1,337 cm^−1^, and 1,391 cm^−1^, respectively. According to Al-Kherssani and Al-Mudhaffar (2024), ZV-AgNPs are synthesized utilizing *Bacillus* sp. The cultures from AW1-2 and their corresponding FTIR spectra demonstrated pronounced peaks at wavenumbers 3433.33 cm^−1^, 2919.36 cm^−1^, 2411.05 cm^−1^, 1625.54 cm^−1^, 1454.23 cm^−1^, and 1128.56 cm^−1^. The presence of a hydroxyl (O–H) group in alcohols is evidenced by the prominent peak observed at wavenumber 3433.33 cm^−1^. Similarly, the vibration corresponding to the C–H group in alkanes is confirmed with the peak at 2919.36 cm^−1^. The absorption bands observed at 2411.05 cm^−1^, 1625.54 cm^−1^, 1454.23 cm^−1^, and 1128.56 cm^−1^ can be attributed to the stretching vibrations of the C=O moiety, alkenes C=C moiety, the N–O stretching in nitro containing compounds, and the C–N moiety in amines, respectively (Al-Kherssani and Al-Mudhaffar, 2024).

The crystalline attributes of the bio-manufactured silver nanoparticles were corroborated through X-ray diffraction (XRD) analysis. The peaks manifesting in the diffraction pattern align with the crystallographic planes, thereby validating the crystalline properties of the material. Crystallite dimensions were derived from the assessment of peak positions, X-ray diffraction data, and full width at half maximum (FWHM), which collectively support the accurate sizing of nanoparticles. This observed crystallinity signifies the effective reduction and stabilization facilitated by *K. flava* KKYHNGU1. The uniformity in crystallite size suggests that the bacterial synthesis method employed is proficient in generating high-quality silver nanoparticles characterized by meticulously organized morphology and dimensions.

Peaks identified at 2θ angles of 37°, 44.8°, 64.9°, and 77° in the investigation conducted by Rajamanickam correspond to the crystallographic planes of 111, 200, 220, and 311, respectively (Govindaraj, 2025). As stated by Daniel et al. (2022), the angles of 2θ measured at 38.11°, 44.3°, 64.4°, and 77.3° correspond to Bragg’s reflections of the crystallographic orientations 111, 200, 220, and 311 correspondingly. The biogenic ZV-AgNPs were found to exhibit an average particle size of 27.31 nm (Govindaraj, 2025).

The scanning electron microscopy (SEM) analysis afforded a definitive representation of uniformly distributed spherical AgNPs. This morphological consistency is advantageous for a myriad of applications. The mean size of the nanoparticles was assessed at 96 nm, indicating enhanced reactivity due to an increased surface area. The evaluation of a 7 mm area at a 400 nm scale under high vacuum conditions set at 20 kV presents a remarkable opportunity for the investigation of the dispersion and morphology of the nanoparticles. The comprehensive analysis concluded that the applied synthesis methodology may yield nanoparticles exhibiting consistent dimensions and morphology. Energy-dispersive spectroscopy (EDS) analysis corroborated the purity of the bio-synthesized AgNPs, revealing a composition of 87.06% silver, 9.69% carbon, 3.25% oxygen, and a complete absence of measurable phosphorus. The highly concentrated silver content underscores the efficacy of the bio-synthesis protocol in producing AgNPs of elevated purity (Weber et al., 2025). The detected carbon and oxygen may originate from bacterial organic extracellular compounds, which could function as capping agents for the AgNPs, thereby enhancing the stability and functional characteristics of the nanoparticles (Jose et al., 2024). The absence of phosphorus implies minimal contamination, thereby reinforcing the purity and quality of the synthesized AgNPs. Consequently, the SEM and EDS analyses substantiate that the bacteriological bio-synthesis mechanism yields superior quality AgNPs distinguished by uniform spherical morphology, appropriate dimensions, and elevated purity standards. Concurrently, Paterlini et al. (2021) noted the observation of sphere-formed AgNPs at a 0.5 μm scale, showcasing sizes between 14 and 92 nm. Using SEM, researchers found that the AgNPs had dimensions ranging from 60 to 80 nm. Not long ago, Jose and colleagues achieved the successful creation of spherical AgNPs derived from *Pseudomonas otitidis*, which displayed an average diameter of 82.76 nm, as revealed by field emission scanning electron microscopy (Jose et al., 2024).

The study’s results indicate that *Staphylococcus aureus* presented the most considerable zone of inhibition, at 14 mm, suggesting its heightened responsiveness to the AgNPs at a level of 3 mg. This observation suggests that AgNPs possess the potential to effectively induce cellular death through their interaction with the bacterial cell membrane. Conversely, *S. marcescens* exhibited a diminished zone of growth inhibition at 10 mm, reflecting a reduced level of sensitivity. The lower antimicrobial activity observed against this Gram-negative bacterium may be ascribed to the makeup of its outer membrane and could additionally be connected to its ability to endure severe environmental circumstances, presenting another barrier to antibacterial initiatives. These bio-fabricated AgNPs have also shown promising results for the other two bacterial strains (*E. coli* and *B. cereus*) used to check antimicrobial potency. Ultimately, this investigation elucidated that AgNPs represent promising active bactericidal agents that exhibit efficacy against Gram-positive as well as Gram-negative bacteria, thereby underscoring their potential applications as broad spectrum antibacterial agent in the medical field. Moreover, the bactericidal potency of AgNPs against various bacterial isolates has been corroborated in several studies (Table 2).

**Table 2:** Biosynthesized AgNPs from various bacterial sources exhibiting antibacterial activity.

S. No.	Source organism(s)	Target organism(s)	References
1	*Lactobacillus* sp., *Bacillus* sp.	*Staphylococcus aureus Pseudomonas aeruginosa*	Al-Asbahi et al. (2024)
2	*Bacillus licheniformis*	*Staphylococcus aureus Pseudomonas aeruginosa*	Karley et al. (2024)
3	*Lactobacillus acidophilus*	*Proteus vulgaris* *Escherichia coli* *Staphylococcus aureus* *Klebsiella pneumoniae*	Khalifa et al. (2023)
4	*Paenibacillus* sp. *MAHUQ-63*	*Salmonella enteritidis*	Huq et al. (2023)
5	*Aggregatimonas sangjinii F202Z8T*	*Escherichia coli* *Bacillus subtilis Staphylococcus aureus*	Kwon et al. (2023)
6	*Microbacterium proteolyticum LA2(R)* *Streptomyces rochei LA2(O)*	*Streptococcus pneumoniae* *Haemophilus influenzae* *Neisseria meningitidis*	Bano et al. (2023)
7	*Escherichia coli VM1*	*Staphylococcus aureus* *E. coli* *Klebsiella pneumoniae* *S. paratyphi* *Vibrio parahaemolyticus* *V. cholera* *Proteus mirabilis*	Sundaramanickam and Maharani (2021)
8	Bacterial culture (SP3)	*Vibrio vulnificus* *Bacillus cereus*	Saritha and Prabha (2022)
9	*L. pentosus S6* *L. plantarum F22* *L. crustorum F11* *L. paraplantarum KM1*	*Staphylococcus aureus*	Sharma et al. (2022)
10	*Viridibacillus* sp.	*E. coli* *P. aeruginosa*	Singh and Mijakovic (2022)

A research investigation focusing on the remediation of trimethoprim utilizing AgNPs demonstrated marked enhancements in efficiency as the antibiotic concentrations diminished, achieving a reduction of 63.42% at 10 ppm, which signifies an effective interaction between trimethoprim and AgNPs, thereby harmonizing the presence of the antibiotic with the reactivity of the nanoparticles. A peak efficiency of 85.40% was attained at a concentration of 10 ppm, suggesting improved interaction due to the augmented availability of functional groups with functional and chemically active sites on the nanoparticles. The maximum absorbance observed at 270 nm confirmed the presence of TMP and exhibited a consistent decrease over time, thereby validating the efficacy of AgNPs in bioremediation of antibiotic. These results underscore the potential of AgNPs as effective agents for the remediation of trimethoprim in aqueous environments, highlighting the necessity to optimize concentration of this anthropogenic compound for effective treatment and reinforcing their important role in mitigating antibiotic contamination in soil and wastewater treatment contexts. Similarly, the study lead by Alishiri et al. (2023) documented 91.23–95.95% removal rate of the TMP antibiotic using magnetite-chitosan NPs at concentration of 22 mg/L. It is essential to point out that this research may be an innovative pursuit in leveraging silver nanoparticles (AgNPs) for extracting trimethoprim from wastewater.

A plethora of scholarly inquiries have been directed towards the evaluation of the anticancer and antitumor properties of silver nanoparticles, with numerous investigations yielding promising results across a wide spectrum of cellular models. The findings from Roy et al. (2024) reveal that silver nanoparticles (AgNPs) are marked by their diminutive size, generally situated in the nanometer spectrum, which aids their entry into malignant cells and nearby tissue. This diminutive scale enhances their ability to interact with cellular components and disrupt cellular processes. As AgNPs penetrate malignant cells, they can stimulate oxidative stress through the release of reactive oxygen species (ROS). The oxidative stress that results can inflict harm on cellular elements such as DNA, proteins, and lipids, ultimately resulting in cell death (apoptosis) (Gomes et al., 2021; Shaaban et al., 2024). This system leads to the activation of pro-apoptotic genes and concurrently blocks anti-apoptotic proteins, including BCL-2 (El-Naggar et al., 2024). These fundamental mechanisms necessitate further exploration, particularly concerning *in vivo* activity and therapeutic efficacy. Recent academic contributions have been summarized in Table 3. Moreover, an extensive appraisal of the IC50 value identified in this research (0.04 mg/mL) has been executed in tandem with ongoing research into the ramifications of AgNPs on assorted cell lines.

**Table 3:** Bio-fabricated AgNPs with anti-cancerous activity.

S. No.	Source organism(s)	Cell line	IC50 (mg/mL)	References
1	*Streptomyces enissocaesilis BS1*	MCF-7 Caco-2	0.160 0.156	Shaaban et al. (2024)
2	*Lactobacillus acidophilus*	HepG2	0.004	Elmetwalli et al. (2024)
3	*Streptomyces* sp. *PG12*	A549 MCF-7	0.069 0.138	Pallavi et al. (2021)
4	*Pseudomonas aeruginosa*	HCC	0.062	Mohammed et al. (2023)
5	*Paenibacillus* sp. *NS-36*	*HCT-116*	0.081	Sreenivasa et al. (2021)
6	*Rhodococcus rhodochrous*	HepG2	0.049	Alam et al. (2021)

## 5 Conclusion

This study establishes *Kocuria flava* KKYHNGU1, isolated from rooftop solar panels, as a novel source for the green synthesis of silver nanoparticles with multifunctional activities. The work highlights the potential of extremophilic microbes from unexplored niches in advancing sustainable nanotechnology. While the results confirm antimicrobial, anticancer, and antibiotic-removal applications, limitations such as particle heterogeneity and incomplete mechanistic insights remain. Future research should focus on detailed physicochemical characterization, mechanistic validation of therapeutic effects, and evaluation of environmental safety to strengthen translational prospects. These directions will help transform solar panel microbiomes into a valuable platform for their biotechnological potential.

## Data Availability

The datasets presented in this study can be found in online repositories. The names of the repository/repositories and accession number(s) can be found in the article/supplementary material.
